# Disproportionality analysis of safety signals for milnacipran and levomilnacipran: a pharmacovigilance study using the FDA adverse event reporting system

**DOI:** 10.3389/fphar.2025.1719881

**Published:** 2025-12-11

**Authors:** Tao Huang, Po Zhang, Yan Zhou, Lei Wang, Qing Zhang, Mingxing Li, Junli Xiao

**Affiliations:** 1 Department of Neurosurgery, Union Hospital, Tongji Medical College, Huazhong University of Science and Technology, Wuhan, Hubei, China; 2 Wuhan Mental Health Center, Wuhan, China; Affiliated Wuhan Mental Health Center, Tongji Medical College of Huazhong University of Science and Technology, Wuhan, Hubei, China

**Keywords:** milnacipran, levomilnacipran, adverse events, FAERS, pharmacovigilance analysis, sex differences

## Abstract

**Background:**

Major depressive disorder (MDD) is a global health concern, with serotonin-norepinephrine reuptake inhibitors (SNRIs) constituting a mainstay of the psychopharmacological approach to its clinical management. Milnacipran and levomilnacipran are SNRIs with distinct serotonin/norepinephrine reuptake ratios. Their real-world adverse event (AE) profiles and sex-specific patterns remain incompletely characterized owing to limitations of small-scale clinical trials.

**Methods:**

Utilizing the FAERS database, adverse event signals were identified for milnacipran (2,752 cases) and levomilnacipran (715 cases) through the methods of Reporting Odds Ratio (ROR), Proportional Reporting Ratio (PRR), Bayesian Confidence Propagation Neural Network (BCPNN), and Multi-Item Gamma Poisson Shrinker (MGPS). Sex-stratified analyses were also conducted, employing a modified ROR approach.

**Results:**

Female patients predominated in AE reports (milnacipran: 79.61%; levomilnacipran: 64.48%). At the level of System Organ Class, milnacipran exhibited significant signals in vascular disorders (EBGM05 = 2.16), while levomilnacipran demonstrated stronger signals in psychiatric (EBGM05 = 3.45), reproductive (EBGM05 = 8.65), and renal/urinary systems (EBGM05 = 2.39). Out of 26 shared Preferred Terms, painful ejaculation and urinary retention showed the largest disparities (levomilnacipran risk higher). Sex-specific signals included female-predominant nausea (milnacipran) and suicidal ideation (levomilnacipran), and male-predominant urinary retention (both agents).

**Conclusion:**

This real-world pharmacovigilance study has highlighted the distinct adverse event profiles of milnacipran and levomilnacipran, with a particular emphasis on sex-specific reactions. This differentiation supports the adoption of precision prescribing strategies and addresses the limitations often encountered in traditional clinical trial data.

## 1 Introduction

Major depressive disorder (MDD) is a prevalent and recurrent psychiatric condition that significantly contributes to global disability. Pharmacological treatments, particularly serotonin-norepinephrine reuptake inhibitors (SNRIs), are the primary options for managing this disorder. Milnacipran and its levorotatory enantiomer, levomilnacipran, are important medications due to their dual inhibition of serotonin (5-HT) and norepinephrine (NE) transporters (Shelton, 2019; Zadka et al., 2016). Milnacipran is approved for the treatment of fibromyalgia in the U.S. and for MDD in Europe and Japan, while levomilnacipran is approved by the U.S. FDA for MDD (Lecrubier et al., 1996; Kamijima et al., 2013).

Despite structural homology, preclinical investigations revealed critical differences in their 5-HT/NE transporter inhibition ratios. Levomilnacipran exhibited higher norepinephrine transporter (NET) affinity than milnacipran, yielding a norepinephrine-preferring 1:2 5-HT/NE reuptake ratio, whereas milnacipran exhibited a balanced 1:1 ratio (Puozzo et al., 2002; Auclair et al., 2013). These chiral-dependent mechanistic disparities may underpin variability in adverse event (AE) profiles (Caillet et al., 2012; Smith, 2009). Clinical trials reported that levomilnacipran most commonly caused nausea (17.1%), dry mouth (10.1%), and constipation (8.5%), whereas milnacipran exhibited a higher incidence of nausea (37%) and vascular risks (e.g., hypertension) (Trugman et al., 2014; Zadka et al., 2016).

However, current evidence mainly derives from small-sample clinical trials (Puech et al., 1997; Mago et al., 2013; Asnis et al., 2013; Montgomery et al., 2013; Sambunaris et al., 2014; Sansone and Sansone, 2014), whose stringent inclusion criteria and limited sample sizes are insufficient to capture the diverse demographic and clinical characteristics of real-world populations. The preliminary clinical findings suggested sex-specific patterns in adverse event distribution, including a higher incidence of gastrointestinal reactions in female patients with milnacipran (Lin et al., 2025) and more frequent urinary retention in male patients with levomilnacipran (Asnis et al., 2016), which also lacked generalizability due to small samples and short follow-up.

Therefore, this study aims to investigate the overall safety profiles of both drugs and validate the pre-specified hypothesis that their chiral-dependent 5-HT/NE inhibition ratios lead to differential AE risks (e.g., norepinephrine-preferring levomilnacipran may exhibit higher cardiovascular/urinary AEs, while milnacipran shows more 5-HT-mediated gastrointestinal reactions).

To address these limitations, the present study aims to systematically analyze the adverse event profiles of milnacipran and levomilnacipran using real-world data from the US FDA Adverse Event Reporting System (FAERS). As one of the world’s largest post-marketing pharmacovigilance databases, FAERS encompasses diverse patient populations, facilitates the capture of rare adverse events, and provides a robust framework for investigating the real-world safety profiles of chiral drugs.

Our study aims to systematically characterize type-specific and sex-stratified adverse event signals associated with milnacipran and levomilnacipran in real-world clinical settings. Additionally, we seek to clarify the role of 5-HT/NE inhibition ratios in explaining the differences in toxicity between these two medications. Lastly, our goal is to establish evidence-based guidelines for individualized therapeutic strategies tailored to specific drugs and sexes.

Beyond validating preliminary findings from clinical trials through real-world evidence, our work aims to identify previously uncharacterized risk profiles, enhance mechanistic insights into the toxicity of chiral drugs, and ultimately refine the safe clinical use of SNRIs in the management of MDD.

## 2 Materials and methods

### 2.1 Data sources and pre-processing

The data for this study originated from the FDA FAERS, a publicly accessible database that aggregates spontaneous adverse event reports globally. This comprehensive database contains seven structured subfiles: demographic and administrative information (DEMO), drug/biologic information (DRUG), adverse event details (REAC), patient outcomes (OUTC), report sources (RPSR), therapy start dates and end dates (THER), and indication information (INDI).

To ensure data reliability, FAERS data extracted from Q1 2004 to Q1 2025 underwent deduplication and cleaning according to FDA-recommended standardized procedures: when CASEID (the number that identifies FAERS cases) was the same, the most recent FDA_DT was selected; when both the CASEID and FDA_DT fields were the same, the report with the higher PRIMARYID was selected. Since Q1 2019, each quarterly data packet included a designated list of CASEIDs corresponding to reports marked for removal. Following data deduplication, reports were systematically excluded based on the CASEIDs enumerated in this list.

Following data cleaning, we performed focused extraction of adverse event reports associated with milnacipran and levomilnacipran, restricting the suspicion level to “primary suspect” within the data files. Subsequently, we extracted key clinical characteristics, encompassing patient demographics (sex, age), reporting region, reporting time, adverse events (AEs), and patient outcomes. Adverse events were systematically encoded using the Medical Dictionary for Regulatory Activities (MedDRA, version 28.0), In the MedDRA system, the Preferred Term (PT) is used to precisely describe specific events, while the System Organ Class (SOC) serves as the highest-level classification, organizing related medical information into a structured hierarchy.

### 2.2 Time to onset analysis

The time to onset (TTO) of milnacipran and levomilnacipran-related AEs was defined as the interval between EVENT_DT (date of AE onset in the DEMO file) and START_DT (date of drug initiation in the THER file). Cases with missing dates (either the initiation of drug therapy or the onset of AEs) or inaccuracies (not specified to a particular day, month, or year) were excluded. Additionally, cases in which the onset date of AE did not occur after the initiation date of drug therapy were also excluded.

### 2.3 Data analysis

We employed four signal detection methods of disproportionality analysis to perform association analysis between drugs and adverse events: the Reporting Odds Ratio (ROR) (Rothman et al., 2004), the Proportional Reporting Ratio (PRR) (Evans et al., 2001), utilized by the UK Medicines and Healthcare products Regulatory Agency (MHRA), the Bayesian Confidence Propagation Neural Network (BCPNN) (Bate et al., 1998), and the Multi-Item Gamma Poisson Shrinker (MGPS) (Dumouchel, 1999). These methods rely on a 2 × 2 contingency table (Table 1) to evaluate the strength of association between drugs and adverse events by computing specific statistical measures. The specific computational formulas and corresponding decision thresholds for each method are provided in Table 2. To minimize false positive signals, this study considered an association indicative of a risk signal related to milnacipran or levomilnacipran only when all four methods exhibited statistical significance.

**TABLE 1:** The fourfold table of disproportionality measurement.

Drug category	Number of target adverse events	Number of other adverse events	Total
Target drug	a	b	a+b
Other drugs	c	d	c + d
Total	a+c	b + d	a+b + c + d

**TABLE 2:** Calculation formulas and detection standards of signal mining.

Method	Formula		Threshold value
ROR	$ROR=\frac{ad}{bc}$		A ≥3 and 95% CI (lower limit) > 1
	$95\%CI=e^{\ln\left(ROR\right)\pm 1.96\sqrt{\left(\frac{1}{a}+\frac{1}{b}+\frac{1}{c}+\frac{1}{d}\right)}}$		
MHRA	$PRR=\frac{a/\left(a+b\right)}{c/\left(c+d\right)}$		A ≥3, PRR ≥2 and *X* ^2^ ≥ 4
	$X^{2}=\frac{{\left(ad‐bc\right)}^{2}\left(a+b+c+d\right)}{\left(a+b\right)\left(c+d\right)\left(a+c\right)\left(b+d\right)}$		
BCPNN	$IC=\log_{2}\frac{a\left(a+b+c+d\right)}{\left(a+b\right)\left(a+c\right)}$		IC025 > 0
	$E\left(IC\right)=\log_{2}\frac{\left(a+\gamma 11\right)\left(a+b+c+d+\alpha \right)\left(a+b+c+d+\beta \right)}{\left(a+b+c+d+\gamma \right)\left(a+b+\alpha 1\right)\left(a+c+\beta 1\right)}$		
	$V\left(IC\right)=\frac{1}{{\left(\ln2\right)}^{2}}\left[\frac{\left(a+b+c+d\right)‐a+\gamma ‐\gamma 11}{\left(a+\gamma 11\right)\left(1+a+b+c+d+\gamma \right)}+\frac{\left(a+b+c+d\right)‐\left(a+b\right)+\alpha ‐\alpha 1}{\left(a+b+\alpha 1\right)\left(1+a+b+c+d+\alpha \right)}+\frac{\left(a+b+c+d\right)‐\left(a+c\right)+\beta ‐\beta 1}{\left(a+c+\beta 1\right)\left(1+a+b+c+d+\beta \right)}\right]$		
	$\gamma =\gamma_{11}\frac{\left(a+b+c+d+\alpha \right)\left(a+b+c+d+\beta \right)}{\left(a+b+\alpha 1\right)\left(a+c+\beta 1\right)}$	α *1* = *β1* = *1;* α = *β* = *2; γ11* = *1*	
	$IC025=E\left(IC\right)‐2\sqrt{V\left(IC\right)}$		
MGPS	$EBGM=\frac{a\left(a+b+c+d\right)}{\left(a+c\right)\left(a+b\right)}$		EBGM05 > 2
	$EBGM05=e^{\ln\left(EBGM\right)‐1.96\sqrt{\left(\frac{1}{a}+\frac{1}{b}+\frac{1}{c}+\frac{1}{d}\right)}}$		

To further investigate the risk differences of AEs by sex, we extracted the relevant data (Table 3). Using a modified ROR signal mining approach, we assessed the influence of sex on the reporting rates of specific AEs. The criteria were as follows: a ROR <1 combined with a chi-square test p-value <0.05 indicated a significantly higher reporting rate for a particular AE among male patients compared to female patients. Conversely, a ROR >1 combined with a chi-square test p-value <0.05 indicated a significantly higher reporting rate for a particular AE among female patients compared to male patients.

**TABLE 3:** The fourfold table for disproportionality analysis of the sex difference in AEs.

Sex	Number of target adverse events	Number of other adverse events	Total
Female	a	b	a+b
Male	c	d	c + d
Total	a+c	b + d	a+b + c + d

Data processing and statistical analysis were performed using Python. GraphPad Prism 8 was employed to generate high-quality graphs for the intuitive presentation of the results.

## 3 Results

### 3.1 The characteristics of milnacipran and levomilnacipran AE reports

Table 4 presents the demographic and clinical characteristics of milnacipran (n = 2,752 cases; 8,657 adverse events) and levomilnacipran (n = 715 cases; 2,081 adverse events) within the FAERS database. In these case reports, female patients were predominant for both drugs (milnacipran: 2,191 cases, 79.61%; levomilnacipran: 461 cases, 64.48%), while males accounted for 13.66% and 28.25%, respectively. The age data for milnacipran and levomilnacipran had substantial missingness (37.68% and 54.41%, respectively). Within the available dataset, the predominant patient population was in the 18–64 age range (milnacipran: 49.67%; levomilnacipran: 36.22%). Similarly, with respect to body weight, both medications exhibited striking data incompleteness, with 81.32% of milnacipran cases and 78.88% of levomilnacipran cases lacking weight recordings. Regarding outcomes, “Other Serious” were reported in 14.93% of milnacipran cases and 21.54% of levomilnacipran cases, while “Death” occurred in 1.82% and 2.52% of cases, respectively.

**TABLE 4:** The characteristics of milnacipran and levomilnacipran case reports (n%).

Characteristics	Milnacipran (n = 2,752)	Levomilnacipran (n = 715)
AE reports number	8,657	2,081
Sex		
Female	2,191 (79.61)	461 (64.48)
Male	376 (13.66)	202 (28.25)
Not specified	185 (6.72)	52 (7.27)
Age (years)		
<18	15 (0.55)	18 (2.52)
18–64	1,367 (49.67)	259 (36.22)
≥65	333 (12.10)	49 (6.85)
Not specified	1,037 (37.68)	389 (54.41)
Weight (kg)		
≤50	27 (0.98)	8 (1.12)
51–100	394 (14.32)	107 (14.97)
> 100	93 (3.38)	36 (5.03)
Not specified	2,238 (81.32)	564 (78.88)
Outcome		
Death	50 (1.82)	18 (2.52)
Life-threatening	70 (2.54)	8 (1.12)
Hospitalization - initial or prolonged	306 (11.12)	71 (9.93)
Disability	34 (1.24)	11 (1.54)
Congenital anomaly	2 (0.07)	0 (0)
Required intervention to prevent permanent impairment/Damage	102 (3.71)	11 (1.54)
Other serious (important medical event)	411 (14.93)	154 (21.54)
Not specified	1,777 (64.57)	442 (61.82)
Reporter		
Physician	810 (29.43)	166 (23.22)
Pharmacist	95 (3.45)	24 (3.36)
Other health - professional	455 (16.53)	59 (8.25)
Lawyer	3 (0.11)	0 (0)
Consumer	1,233 (44.80)	420 (58.74)
Not specified	156 (5.67)	46 (6.43)
Report year		
2009	487 (17.70)	0 (0)
2010	736 (26.74)	0 (0)
2011	449 (16.32)	0 (0)
2012	140 (5.09)	0 (0)
2013	188 (6.83)	2 (0.28)
2014	61 (2.22)	105 (14.69)
2015	53 (1.93)	120 (16.78)
2016	86 (3.12)	112 (15.66)
2017	127 (4.61)	120 (16.78)
2018	111 (4.03)	57 (7.97)
2019	66 (2.40)	60 (8.39)
2020	81 (2.94)	28 (3.92)
2021	30 (1.09)	37 (5.17)
2022	36 (1.31)	33 (4.62)
2023	48 (1.74)	27 (3.78)
2024	32 (1.16)	11 (1.54)
2025	21 (0.76)	3 (0.42)
Time to event onset (days)		
≤30	479 (17.41)	47 (6.57)
31–180	153 (5.56)	21 (2.94)
181–360	42 (1.53)	6 (0.84)
≥361	55 (2.00)	4 (0.56)
Not specified	2,023 (73.51)	637 (89.09)
Dose (mg/daily)		
≤20	183 (6.65)	40 (5.59)
21–50	502 (18.24)	30 (4.20)
51–100	394 (14.32)	35 (4.90)
101–200	60 (2.18)	10 (1.40)
≥201	7 (0.25)	0 (0)
Not specified	1,606 (58.36)	600 (83.92)

Consumers were the primary reporters (milnacipran: 44.80%; levomilnacipran: 58.74%), followed by physicians (milnacipran: 29.43%; levomilnacipran: 23.22%). The number of milnacipran case reports peaked in the early 2010s, particularly in 2010, before undergoing a substantial decline by 2014, and stabilized at consistently low levels through 2025. Levomilnacipran case reports reached high volumes from 2014 to 2017 but subsequently decreased steadily to low levels in the later years.

Regarding the time to event onset, the majority of reports for both medications (milnacipran: 2023 cases, 73.51%; levomilnacipran: 637 cases, 89.09%) lacked the specified onset timing. Among the reports with available data, milnacipran showed the highest proportion of adverse events occurring within 30 days (17.41%, 479 cases), followed by 31–180 days (5.56%, 153 cases) and 361 days or more (2.00%, 55 cases). For levomilnacipran, the most frequently specified onset period was also ≤30 days (6.57%, 47 cases), with smaller proportions reported for other intervals. Notably, dose data also had high missing rates (milnacipran: 1,606 cases, 58.36%; levomilnacipran: 600 cases, 83.92%).

### 3.2 SOC level disproportionality analysis of milnacipran and levomilnacipran

**FIGURE 1:**
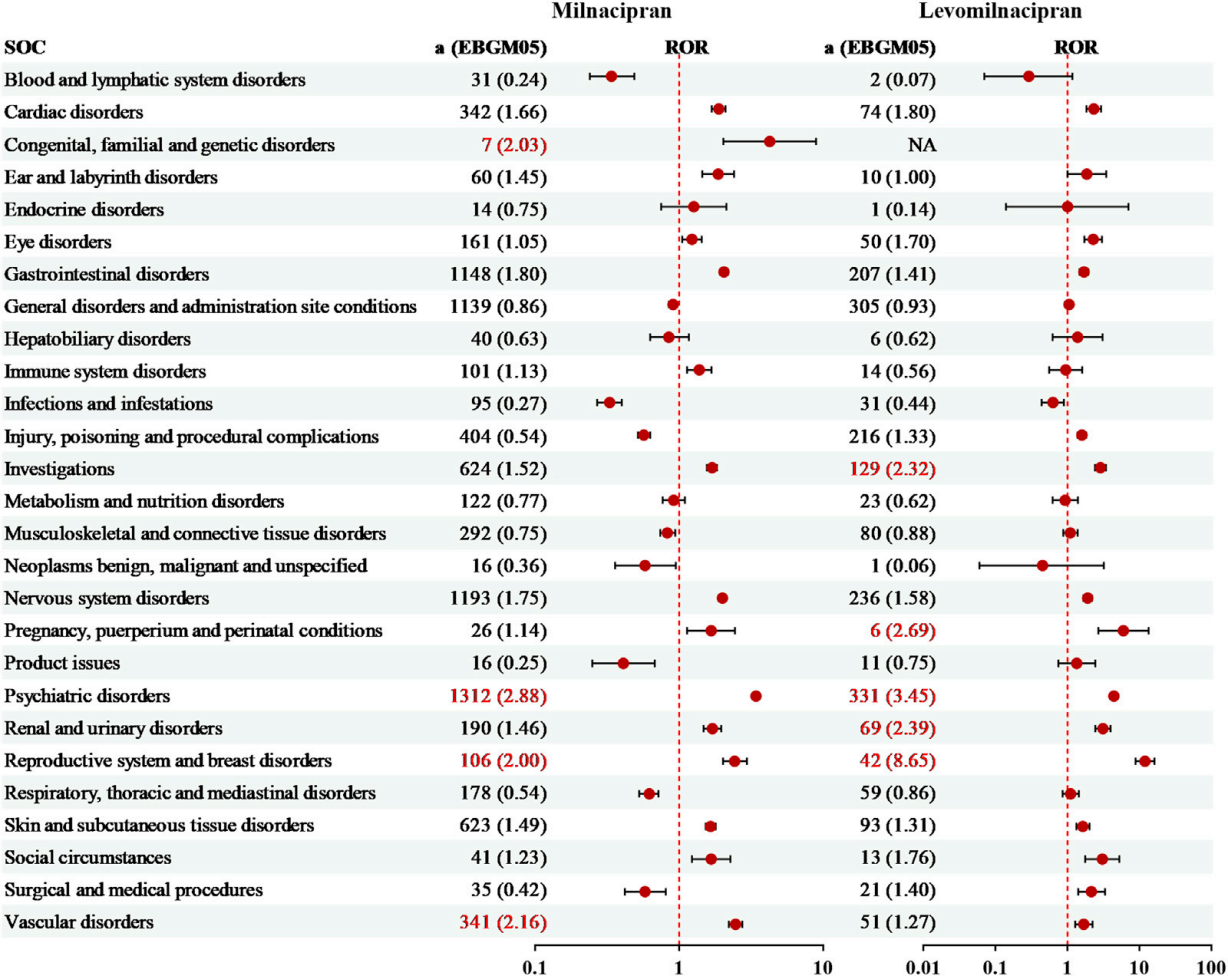
The signal strengths of adverse events of milnacipran and levomilnacipran at the SOC level. Red numbers denote significant risk signals.

The AEs associated with milnacipran impacted 27 organ systems, whereas levomilnacipran affected 26 organ systems (Figure 1). Except for the congenital, familial, and genetic disorders, exclusively associated with milnacipran (7 cases, EBGM05 = 2.03), the 26 System Organ Classes (SOCs) affected by both drugs showed complete overlap. Milnacipran demonstrated 4 SOCs with significant risk signals, while levomilnacipran identified 5 SOCs, all of which met statistical criteria via disproportionality analyses, including ROR, MHRA, BCPNN, and MGPS.

Notably, the largest numbers of AEs were observed in the psychiatric disorders in both milnacipran (1,312 cases, EBGM05 = 2.88) and levomilnacipran (331 cases, EBGM05 = 3.45). The reproductive system and breast disorders, both milnacipran (106 cases, EBGM05 = 2.00) and levomilnacipran (42 cases, EBGM05 = 8.65), also exhibited significant signals. Additionally, milnacipran exhibited significant signals in the vascular disorders (341 cases, EBGM05 = 2.16), whereas levomilnacipran exhibited significant signals in the investigations (129 cases, EBGM05 = 2.32), the pregnancy, puerperium and perinatal conditions (6 cases, EBGM05 = 2.69), and the renal and urinary disorders (69 cases, EBGM05 = 2.39).

### 3.3 The shared risk signals between milnacipran and levomilnacipran

**FIGURE 2:**
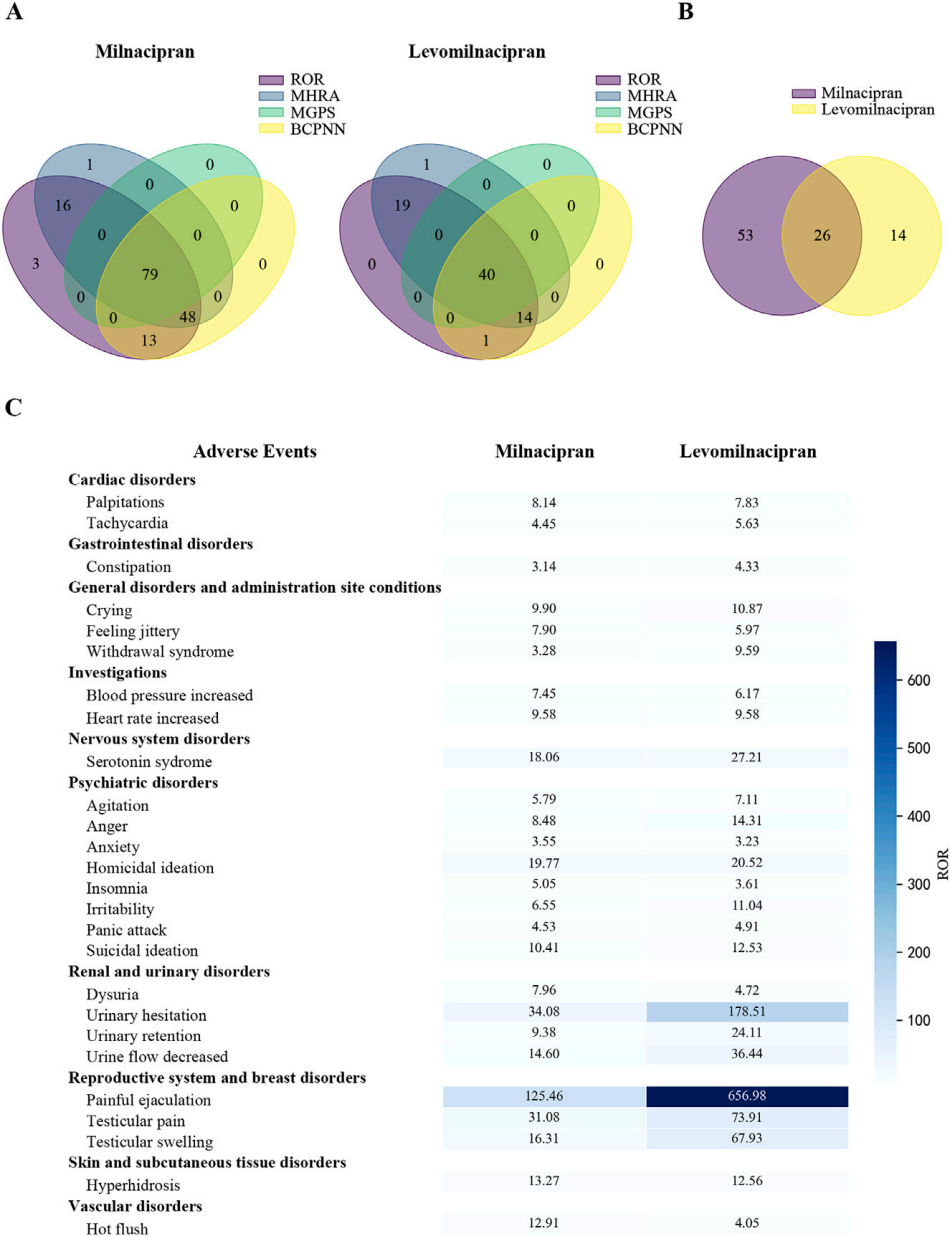
Comparison of significant risk signals between milnacipran and levomilnacipran AEs. **(A)** The Venn diagram under four different algorithms. **(B)** The Venn analysis between milnacipran and levomilnacipran. **(C)** The shared risk signals of the two drugs.

Risk signals associated with drug-induced adverse events were displayed by 79 PTs for milnacipran and 40 PTs for levomilnacipran, as calculated using four algorithms (Figure 2A). Comparing significant risk signals revealed 26 shared PTs between milnacipran and levomilnacipran. Milnacipran showed 53 unique PTs, while levomilnacipran had 14 unique PTs (Figure 2B).

The shared risk signals for milnacipran and levomilnacipran primarily involved ten system organ classes: (1) cardiac disorders (palpitations, tachycardia); (2) gastrointestinal disorders (constipation), (3) general disorders and administration site conditions (crying, feeling jittery, withdrawal syndrome), (4) investigations (blood pressure increased, heart rate increased), (5) nervous system disorders (serotonin syndrome), (6) psychiatric disorders (agitation, anger, anxiety, homicidal ideation, insomnia, irritability, panic attack, suicidal ideation), (7) renal and urinary disorders (dysuria, urinary hesitation, urinary retention, urine flow decreased), (8) reproductive system and breast disorders (painful ejaculation, testicular pain, testicular swelling), (9) skin and subcutaneous tissue disorders (hyperhidrosis), (10) and vascular disorders (hot flush). Among these, two PTs exhibited powerful risk signals: painful ejaculation (milnacipran: 4 cases, ROR = 125.46; levomilnacipran: 5 cases, ROR = 656.98) and urinary hesitation (milnacipran: 12 cases, ROR = 34.08; levomilnacipran: 15 cases, ROR = 178.51) (Figure 2C).

### 3.4 The exclusive risk signals of milnacipran and levomilnacipran

**FIGURE 3:**
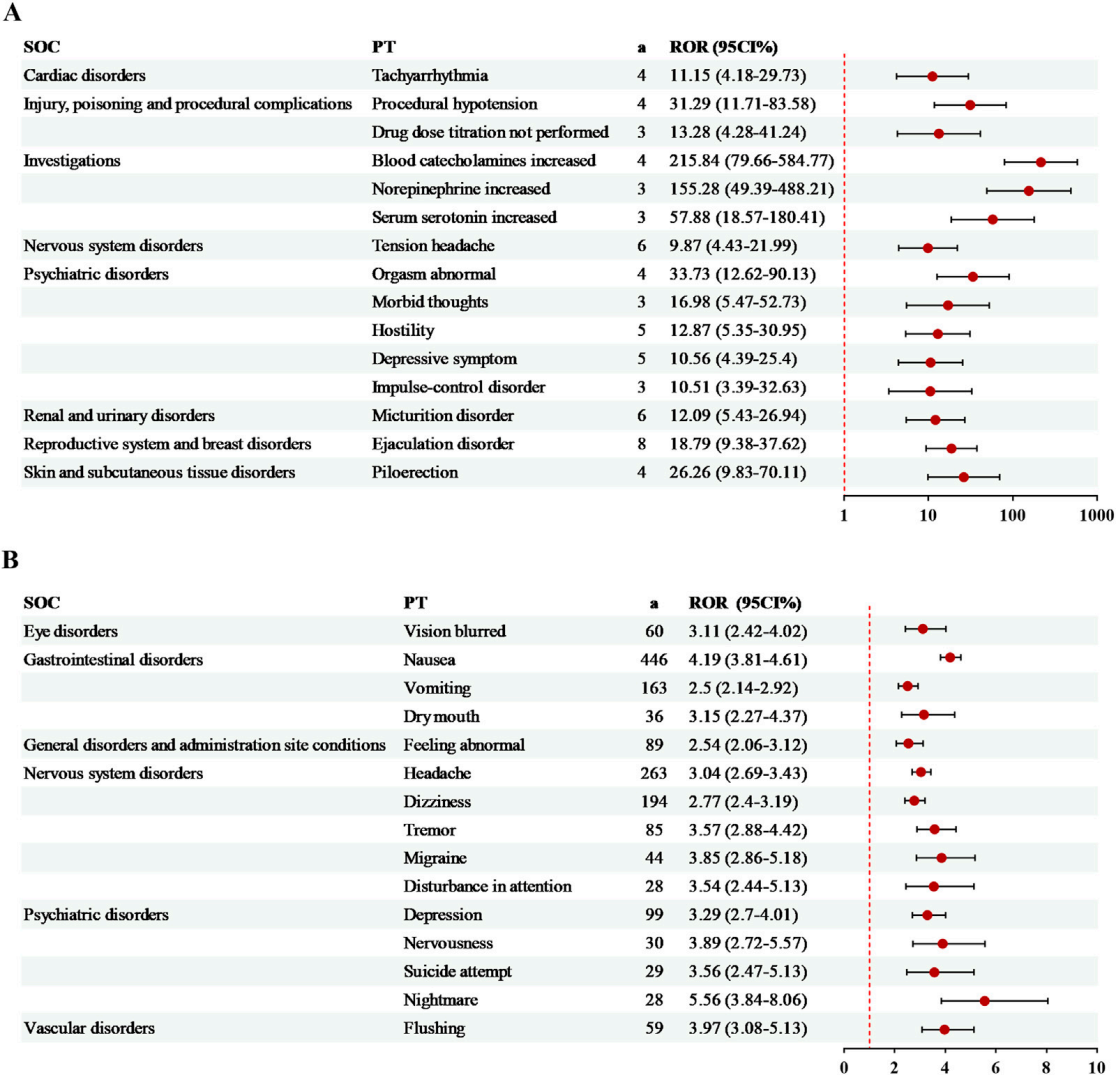
Specific risk signals of milnacipran. **(A)** The top 15 risk signals ranked by the number of reports. **(B)** The top 15 risk signals ranked by ROR. a, number of reports.

The exclusive risk signals of milnacipran were ranked by AE count (Figure 3A) or ROR value (Figure 3B), with the top 15 signals selected for visualization in each case. Among the most frequent AEs were nausea (446 cases, ROR = 4.19), headache (263 cases, ROR = 3.04), and dizziness (194 cases, ROR = 2.77). Additionally, three AEs exhibited relatively high ROR values: blood catecholamines increased (4 cases, ROR = 215.84), norepinephrine increased (3 cases, ROR = 155.28), and serum serotonin increased (3 cases, ROR = 57.88). Notably, several unlabeled signals related to milnacipran were identified, including disturbance in attention, nightmare (Figure 3A), and piloerection (Figure 3B).

**FIGURE 4:**
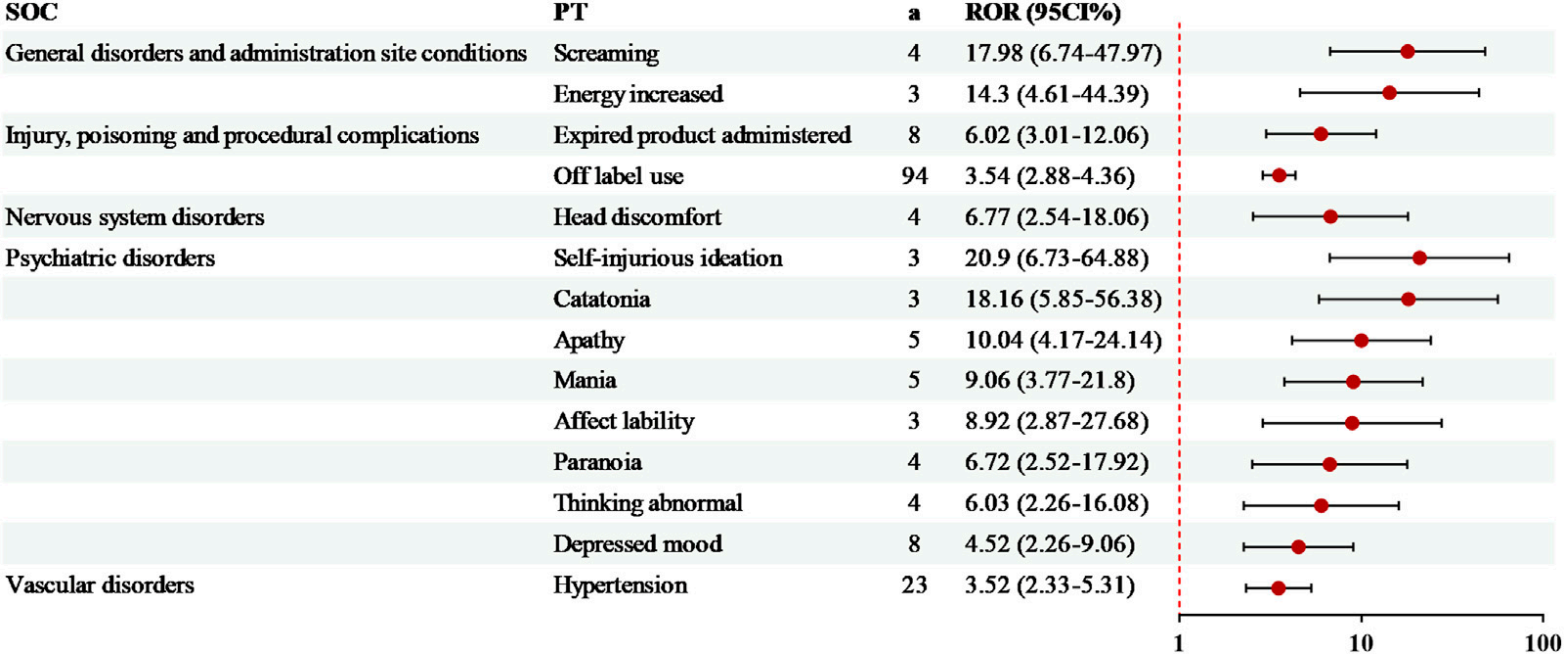
Specific risk signals of levomilnacipran. a, number of reports.

14 exclusive risk signals of levomilnacipran were presented in Figure 4. Among these, the risk signals with a relatively high number of AEs included off-label use (94 cases, ROR = 3.54) and hypertension (23 cases, ROR = 3.52). Additionally, several significant AEs related to levomilnacipran not mentioned in the drug label were identified, such as screaming and energy increased (Figure 4).

### 3.5 Sex difference in AEs related to milnacipran and levomilnacipran

Subsequently, we investigated sex differences in risk signals associated with milnacipran and levomilnacipran by selecting AEs reported in both sexes for statistical analysis. Using the modified ROR method, female patients were identified as having an increased propensity to report specific AEs when the lower bound of the 95% CI for the ROR exceeded 1, with the converse observed in male patients.

For milnacipran (Table 5), the high-risk signals in females included nausea, headache, hot flush, asthenia, and vision blurred. The high-risk signals in males included off-label use, dysuria, urinary retention, death, completed suicide, libido decreased, rash maculo-papular, urinary incontinence, cardiac failure congestive, micturition urgency, pollakiuria, feeling drunk, micturition disorder, urinary hesitation, neuroleptic malignant syndrome, prescribed overdose, sexual dysfunction, and urine flow decreased.

**TABLE 5:** Sex differences in risk signal detection for milnacipran.

SOC	PT	Female/Male	ROR (95% CI)	p value	Direction
Gastrointestinal disorders	Nausea	396/26	2.59 (1.73–3.87)	<0.01	F
Nervous system disorders	Headache	226/20	1.88 (1.19–2.99)	<0.01	F
Vascular disorders	Hot flush	124/7	2.94 (1.37–6.31)	<0.01	F
General disorders and administration site conditions	Asthenia	60/3	3.30 (1.03–10.55)	<0.05	F
Eye disorders	Vision blurred	57/2	4.71 (1.15–19.31)	<0.05	F
Injury, poisoning and procedural complications	Off label use	79/25	0.51 (0.33–0.81)	<0.01	M
Renal and urinary disorders	Dysuria	10/32	0.05 (0.02–0.10)	<0.01	M
Renal and urinary disorders	Urinary retention	10/33	0.05 (0.02–0.10)	<0.01	M
General disorders and administration site conditions	Death	7/6	0.19 (0.06–0.57)	<0.01	M
Psychiatric disorders	Completed suicide	5/8	0.10 (0.03–0.31)	<0.01	M
Psychiatric disorders	Libido decreased	5/6	0.14 (0.04–0.45)	<0.01	M
Skin and subcutaneous tissue disorders	Rash maculo-papular	5/4	0.20 (0.05–0.76)	<0.05	M
Renal and urinary disorders	Urinary incontinence	4/6	0.11 (0.03–0.39)	<0.01	M
Cardiac disorders	Cardiac failure congestive	3/4	0.12 (0.03–0.55)	<0.05	M
Renal and urinary disorders	Micturition urgency	3/5	0.10 (0.02–0.41)	<0.01	M
Renal and urinary disorders	Pollakiuria	3/4	0.12 (0.03–0.55)	<0.05	M
General disorders and administration site conditions	Feeling drunk	2/4	0.08 (0.01–0.45)	<0.01	M
Renal and urinary disorders	Micturition disorder	2/4	0.08 (0.01–0.45)	<0.01	M
Renal and urinary disorders	Urinary hesitation	2/10	0.03 (0.01–0.15)	<0.01	M
Nervous system disorders	Neuroleptic malignant syndrome	1/3	0.05 (0.01–0.53)	<0.05	M
Injury, poisoning and procedural complications	Prescribed overdose	1/3	0.05 (0.01–0.53)	<0.05	M
Reproductive system and breast disorders	Sexual dysfunction	1/3	0.05 (0.01–0.53)	<0.05	M
Renal and urinary disorders	Urine flow decreased	1/4	0.04 (0.00–0.37)	<0.01	M

For levomilnacipran (Table 6), the high-risk signals in females included suicidal ideation and feeling abnormal, whereas the high-risk signals in males included urinary retention, completed suicide, urinary hesitation, and dysuria.

**TABLE 6:** Sex differences in risk signal detection for levomilnacipran.

SOC	PT	Female/Male	ROR (95% CI)	p value	Direction
Psychiatric disorders	Suicidal ideation	35/4	3.33 (1.18–9.41)	<0.05	F
General disorders and administration site conditions	Feeling abnormal	23/2	4.35 (1.02–18.53)	<0.01	F
Renal and urinary disorders	Urinary retention	2/24	0.03 (0.01–0.13)	<0.05	M
Psychiatric disorders	Completed suicide	2/5	0.15 (0.03–0.77)	<0.05	M
Renal and urinary disorders	Urinary hesitation	1/14	0.03 (0.00–0.20)	<0.01	M
Renal and urinary disorders	Dysuria	1/5	0.07 (0.01–0.64)	<0.01	M

**FIGURE 5:**
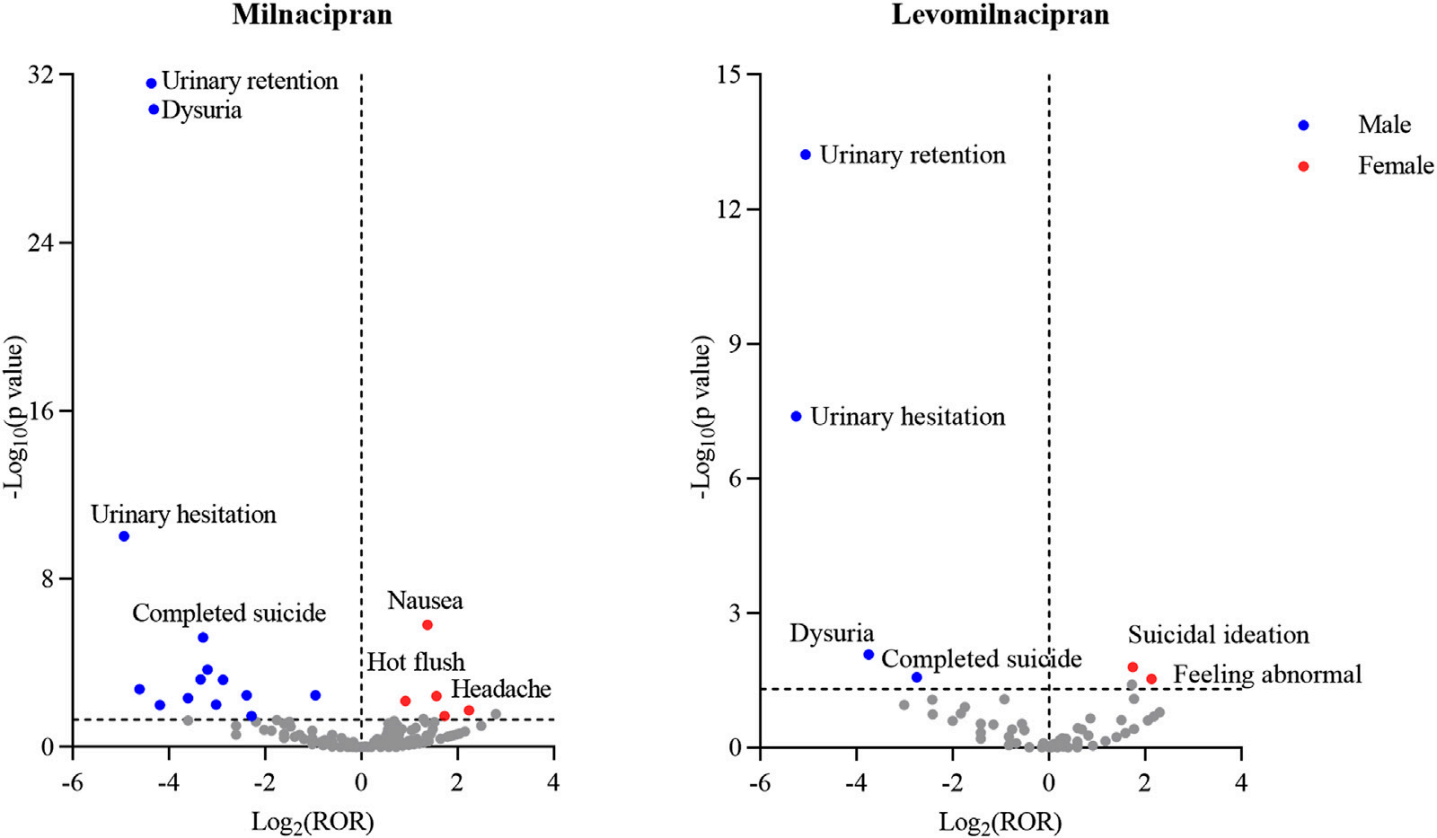
Sex-differentiated risk signal volcano plots for milnacipran and levomilnacipran. The horizontal coordinate showed the Log_2_ (ROR) value and the vertical coordinate indicates the -Log_10_ (p value).

To visually illustrate the sex-specific disparities, volcano plots comparing risk signals between males and females of milnacipran and levomilnacipran were presented (Figure 5). Each data point represents an AE, with statistically significant signals labeled for clarity. Blue dots denote male-predominant potential risk signals, while red dots indicate female-predominant signals.

## 4 Discussion

### 4.1 Analysis of the basic characteristics of milnacipran and levomilnacipran AE reports

Our analysis of adverse event reports for milnacipran (2,752 cases) and levomilnacipran (715 cases) identified several safety characteristics (Table 4). Firstly, although the age data of milnacipran and levomilnacipran AE reports were incomplete, available records indicated that most patients were aged 18–64 years (49.67% and 36.22%, respectively), reflecting the typical adult population treated with these drugs (Fayaz et al., 2016; Lu et al., 2021). Secondly, the distribution of reporters revealed a striking dominance of consumer-driven reporting for both milnacipran (44.80%) and levomilnacipran (58.74%). The high proportion of consumer reports may reflect increased patient engagement in monitoring their own adverse reactions. Thirdly, the temporal trends for milnacipran and levomilnacipran case reports exhibited a similar pattern, with high reporting volumes early on, followed by a decrease and eventual stabilization at low levels. This pattern may indicate a decline in usage after their initial marketing peaks, with factors such as market substitutions or regulatory measures further contributing to the stabilization of their reporting volumes at these lower levels in later periods.

### 4.2 NET/SERT inhibition differences and adverse event profiles

Using data from the FAERS database, our study is the first to demonstrate that milnacipran (racemate) and levomilnacipran (levorotatory isomer) exhibit marked type-specific and sex-specific differences in their adverse event profiles in a real-world setting. This finding is consistent with previous clinical trial results. Furthermore, this large-scale real-world data study addresses the limitations of clinical trials and advances the mechanistic understanding of chiral drug toxicity.

The differential adverse reaction profiles of milnacipran and levomilnacipran are primarily attributed to the distinct ratios of norepinephrine transporter (NET) to serotonin transporter (SERT) inhibition between the two drugs. Levomilnacipran exhibits a markedly higher affinity for NET than milnacipran, while its affinity for SERT is comparable to that of milnacipran *in vitro* (Zadka et al., 2016). Specifically, levomilnacipran demonstrates significantly greater selective inhibition of NET, with a 5-HT: NE inhibitory ratio of 1:2, compared to the balanced 1:1 ratio of milnacipran (Puozzo et al., 2002; Auclair et al., 2013).

This difference may contribute to levomilnacipran exhibiting markedly stronger risk signals than milnacipran in the psychiatric, reproductive, and renal/urinary systems at the SOC level (Figure 1). Specifically, the psychiatric and reproductive system risk signals were notably higher, while renal/urinary system signals were significant, a feature not observed with milnacipran (Figure 1). These observations align with the toxicological mechanism of enhanced NET inhibition leading to noradrenergic hyperactivity (Stahl et al., 2005). In contrast, due to its balanced 5-HT/NE inhibitory profile, milnacipran not only showed more pronounced risks in the vascular and gastrointestinal systems (Figure 1) but also had a greater number of specific adverse events (Figures 2, 3), further supporting the influence of 5-HT/NE ratio differences on peripheral effects (Behlke et al., 2020).

Consistent with previous findings (Zadka et al., 2016), milnacipran exhibited a higher number of risk signals for drug-induced AEs than levomilnacipran, with 79 and 40 PTs (Figure 2), respectively. Among the 26 shared PTs between milnacipran and levomilnacipran, painful ejaculation and urinary retention showed the most marked differences in risk signal intensity, with levomilnacipran being higher than milnacipran for both (Figure 2). This observation aligns with the theory proposed by Asnis (Asnis et al., 2016), whose research suggests that antidepressants that particularly potentiate norepinephrine may be more likely than other antidepressants to induce urinary hesitancy and retention. Additionally, levomilnacipran caused fewer gastrointestinal symptoms and increased the risk of sexual dysfunction in patients (Figures 2, 4), findings supported by the randomized controlled trials (Asnis et al., 2013; Bakish et al., 2014; Gommoll et al., 2014; Sambunaris et al., 2014; Shiovitz et al., 2014).

### 4.3 Sex differences in adverse events

For milnacipran, females accounted for 79.61% of adverse event reports (Table 4), with high-risk symptoms predominating in somatic reactions, including nausea and headache (Table 5). For levomilnacipran, females accounted for 64.48% of adverse event reports (Table 4), with high-risk symptoms characterized by psychiatric reactions such as suicidal ideation (Table 6). The epidemiological studies have demonstrated a higher proportion of female than male patients with depression and fibromyalgia (GBD Mental Disorders Collaborators, 2022; Lu et al., 2021), which may contribute to a relatively greater number of adverse reactions in females receiving medications treatment. Additionally, the primary reporters of AEs were consumers, and the social factors may contribute to females being more proactive in reporting adverse reactions (Lee et al., 2023).

Sex specificity in adverse events may arise from the synergistic effects of anatomical characteristics and receptor regulation (Kim et al., 2016). Case reports of antidepressant-induced urinary hesitancy/retention have mainly been reported in middle-aged and older males, frequently with a history of prostatism (Asnis et al., 2016; Aggarwal et al., 2010; Kasper, 2002; Kasper and Wolf, 2002). However, antidepressant-induced urinary hesitancy/retention also occurs in younger males (Kasper, 2002; Kasper and Wolf, 2002). Despite the age-related predisposition to urinary hesitancy/retention in males (Chughtai et al., 2016), antidepressant-induced urologic signals remain strikingly prominent in men. This male predominance arises because the core mechanism (noradrenergic drugs induce urethral constriction by stimulating α-adrenergic receptors in the smooth muscle of the male prostate gland) drives the gender difference (Kim et al., 2016; Verhamme et al., 2008). Consistently, in our study, urologic adverse events associated with both milnacipran and levomilnacipran were primarily observed in male patients (Figure 5).

### 4.4 Individualized medication recommendations and novel safety signal discovery

Given real-world data and these mechanistic differences, individualized approaches to medication selection are recommended. For patients at high risk of urologic events, milnacipran exhibited a marginally higher safety profile compared to levomilnacipran (Asnis et al., 2016). For patients with MDD presenting with amotivational symptoms, levomilnacipran may be favored, given its enhanced noradrenergic activity, which significantly improved motivational deficits (Gautam et al., 2019). Female patients on milnacipran should undergo close monitoring for 5-HT_3_ receptor-related symptoms (e.g., nausea), with concomitant use of 5-HT_3_ antagonists (e.g., ondansetron) recommended to alleviate these symptoms (Kelly et al., 2008). In male patients who need levomilnacipran, assessment of prostate health is necessary before medication initiation (Asnis et al., 2016).

Beyond offering clinical guidance for individualized treatment, this study addresses the limitations of clinical trials by utilizing its large-sample FAERS dataset. This research is the first to identify previously unlabeled signals. These include screaming and energy increased specific to levomilnacipran, as well as nightmare and piloerection specific to milnacipran. These findings provide a tool for detecting rare toxicities of chiral drugs.

### 4.5 Limitation

Weight data were missing in over 75% of cases (milnacipran: 81.32%; levomilnacipran: 78.88%), which limited the ability to directly analyze the correlation between weight and adverse events. A substantial missingness in outcome data for milnacipran and levomilnacipran limited a comprehensive assessment of the safety profile. Although levomilnacipran showed a slightly higher proportion of “Death” (2.52% vs. 1.82%) compared to milnacipran, “Death” cases may be linked to underlying disease severity rather than direct drug toxicity.

In terms of treatment context, the time to onset of AEs showed a striking pattern: 73.51% of milnacipran and 89.09% of levomilnacipran reports lacked TTO data, but available records indicated that 17.41% of milnacipran AEs and 6.57% of levomilnacipran AEs occurred within 30 days of initiation. This suggests that early monitoring within the first month is critical, particularly for noradrenergic-related events such as urinary retention (Asnis et al., 2016). A small subset of AEs (milnacipran: 2.00%; levomilnacipran: 0.56%) occurred after ≥361 days, highlighting the need for long-term safety surveillance, especially in patients with chronic conditions.

Previous investigations have documented distinct dose-dependent profiles for AEs associated with milnacipran and levomilnacipran. For milnacipran, specific AEs, such as dysuria and cardiovascular reactions, exhibit a clear dose-response relationship, with higher incidence rates at increased doses. In contrast, the AEs of levomilnacipran show a mild dose-related trend; nevertheless, increased exposure may still enhance the risks for certain AEs (Shelton, 2019). However, the current study was unable to investigate these dose-dependent associations due to the frequent absence of detailed dosage data for both drugs (milnacipran: 58.36%; levomilnacipran: 83.92%). This significant gap prevented the assessment of dose and AE correlations, limiting our understanding of how therapeutic dosing affects AE risk profiles.

Beyond the limitations mentioned above, the FAERS database, as a voluntary passive reporting system, has inherent constraints related to the clinical context. Key variables critical for interpreting adverse event associations (such as disease severity, polypharmacy regimens, treatment duration, and medication sequencing) are often insufficiently documented or entirely missing. These omissions hinder the ability to disentangle drug-specific effects from confounders, such as concurrent medications or underlying disease progression, which may independently contribute to adverse event occurrence. Caution is thus warranted when interpreting results, and prospective studies with standardized data collection (encompassing dose, treatment duration, and comprehensive patient covariates) are necessary to validate real-world safety patterns and refine risk stratification. Despite these limitations, the FAERS database provides valuable resources for post-marketing safety monitoring of milnacipran and levomilnacipran.

## 5 Conclusion

In summary, the pharmacovigilance analysis was conducted using the FAERS database to explore AE profiles and sex differences of milnacipran and levomilnacipran, which revealed the disparities in AE patterns between the two drugs and across patient sexes. Male patients exhibited a higher propensity for severe urological events, while female patients were more susceptible to somatic reactions and psychiatric symptoms. The results provide critical insights for healthcare providers to optimize individualized treatment strategies based on sex and drug-specific risks, thereby reducing the incidence of serious AEs. Although the sex-specific AE signals require further validation in prospective cohorts, the findings underscore the value of real-world data in complementing the limitations of clinical trials and advancing the safety monitoring of chiral drugs.

## Data Availability

Publicly available datasets were analyzed in this study. This data can be found here: https://fis.fda.gov/extensions/FPD-QDE-FAERS/FPD-QDE-FAERS.html.
